# Protection against live rotavirus challenge in mice induced by parenteral and mucosal delivery of VP6 subunit rotavirus vaccine

**DOI:** 10.1007/s00705-015-2461-8

**Published:** 2015-05-29

**Authors:** Suvi Lappalainen, Ana Ruth Pastor, Maria Malm, Vanessa López-Guerrero, Fernando Esquivel-Guadarrama, Laura A. Palomares, Timo Vesikari, Vesna Blazevic

**Affiliations:** Vaccine Research Center, University of Tampere Medical School, Biokatu 10, 33520 Tampere, Finland; Instituto de Biotecnología, Universidad Nacional Autónoma de México, Cuernavaca, Morelos México; Facultad de Medicina, Universidad Autónoma del Estado de Morelos, Cuernavaca, Morelos México

**Keywords:** Rotavirus, VP6, IgA, Intranasal, Intramuscular, Protection

## Abstract

Live oral rotavirus (RV) vaccines are part of routine childhood immunization but are associated with adverse effects, particularly intussusception. We have developed a non-live combined RV – norovirus (NoV) vaccine candidate consisting of human RV inner-capsid rVP6 protein and NoV virus-like particles. To determine the effect of delivery route on induction of VP6-specific protective immunity, BALB/c mice were administered a vaccine containing RV rVP6 intramuscularly, intranasally or a combination of both, and challenged with murine RV. At least 65 % protection against RV shedding was observed regardless of delivery route. The levels of post-challenge serum VP6-specific IgA titers correlated with protection.

Rotavirus (RV) causes severe gastroenteritis in infants and children under 5 years of age with high mortality and morbidity rates [1]. Currently, two live oral RV vaccines, the monovalent Rotarix^®^ (GlaxoSmithKline) and the pentavalent Rotateq^®^ (Merck), are licensed and used extensively [2, 3]. However, these oral vaccines are less efficacious in developing countries [4, 5] and are associated with safety concerns such as a risk of intussusception [6]. Non-live subunit RV vaccines are therefore considered as alternatives for RV immunization.

Correlates of protection against RV infection are not fully understood. Type-specific neutralizing antibodies against the external proteins VP4 and VP7 have a role in protective immunity after natural RV infection [7, 8], but their role in vaccine-induced protective immunity against severe RV gastroenteritis has not been shown. Although serum anti-RV antibody IgA titers as a correlate of protection have been disputed [9], the best surrogate marker for RV vaccine-induced protection appears to be a high level of serum RV IgA antibody targeted to the inner capsid protein VP6 [10, 11], which determines viral group (A-H) and subgroup (SGI, II, I+II, non-I/II for group A) specificity [12] and is highly conserved [13], immunogenic [14, 15] and the most abundant RV protein [12]. VP6 does not induce classical neutralizing antibodies, but it induces heterotypic cross-reactive protection in mice [16–18].

Norovirus (NoV) is another leading cause of acute gastroenteritis in children, with genogroups GI and GII being responsible for the majority of NoV cases [19]. For protection against childhood gastroenteritis, we have introduced a concept of vaccination against RV and NoV with a combined trivalent vaccine consisting of RV rVP6 protein and NoV GI.3 and GII.4 virus-like particles (VLPs) [20]. We have previously shown that a candidate combination vaccine delivered intramuscularly (IM) to mice was highly immunogenic [20], and intranasal (IN) immunization protected mice against murine RV challenge [21]. Delivery requirements for the NoV components in the induction of protective NoV immune response were published recently [22]. In this work, we compared IM and IN delivery and the combination of both for induction of VP6-specific protective immunity against RV challenge, and we examined humoral immune responses for correlation with protection.

Human RV rVP6 protein (SGII) used for immunization and as antigen in ELISA was produced using a baculovirus expression system in Sf9 insect cells [23]. The trivalent RV-NoV combination vaccine was prepared by mixing the rVP6 tubules and NoV GI.3 and GII.4 VLPs in equal amounts [20].

Female 7-week-old BALB/c OlaHsd mice (5 mice/group) (Harlan, Horst, The Netherlands) were immunized IM or IN twice (at study weeks 0 and 3) with the trivalent vaccine containing 10 µg or RV rVP6 per immunization point. Moreover, sequential IM and IN immunizations (4 mice/group) with 10 µg of rVP6 alone were performed to determine whether administration at two distinct sites would enhance protection. No external adjuvants were used. Naïve mice receiving PBS served as controls. Pre-immune (week 0) and pre-challenge (week 5) tail blood samples of individual mice were collected, processed to obtain sera and diluted 1:100 in PBS. At week 6, mice were challenged orally with 1 × 10^4^ focus-forming units (FFU) (100 times the diarrheal dose DD_50_) of the murine RV strain EDIM_wt_ (SG non-I/II, G3P10[16]), originally obtained from Dr. Ward (Gamble Institute of Medical Research, Cincinnati, OH). Fecal samples were collected prior to challenge (day 0) and daily for 8 days (days 1-8) after the challenge. Mice were euthanized at day 8, when whole blood samples were also collected. The protocol for the study (permission number 167-2010) was approved by the Bioethics Committee of the Instituto de Biotecnologia (Universidad Nacional Autónoma de México).

RV VP6-specific pre- and post-challenge antibody responses were determined by measuring levels of anti-VP6 IgG and IgA in individual sera at 1:100 and two-fold dilution series by ELISA according to previously published procedures [20, 21].

The presence of RV antigen in fecal samples was determined using an antigen ELISA [16]. Fecal antigen shedding was expressed as the net OD_405_ value (the OD of the pre-challenge fecal sample subtracted from the OD of the post-challenge samples of the individual mouse).

The pre-immune sera of all mice were negative for anti-VP6 IgG and IgA (data not shown). Robust systemic IgG responses were induced by each immunization route (Fig. 1a). Geometric mean titers (GMTs) of serum IgG achieved by the IM, IN and IM+IN routes were equivalent (*p* = 0.663). IN and IM+IN delivery elicited detectable IgA antibodies (*p* = 0.556), while IM immunization did not (Fig. 1b). No anti-VP6 antibodies were detected in sera of control mice prior to the challenge (Fig. 1a and b).

**Fig. 1:**
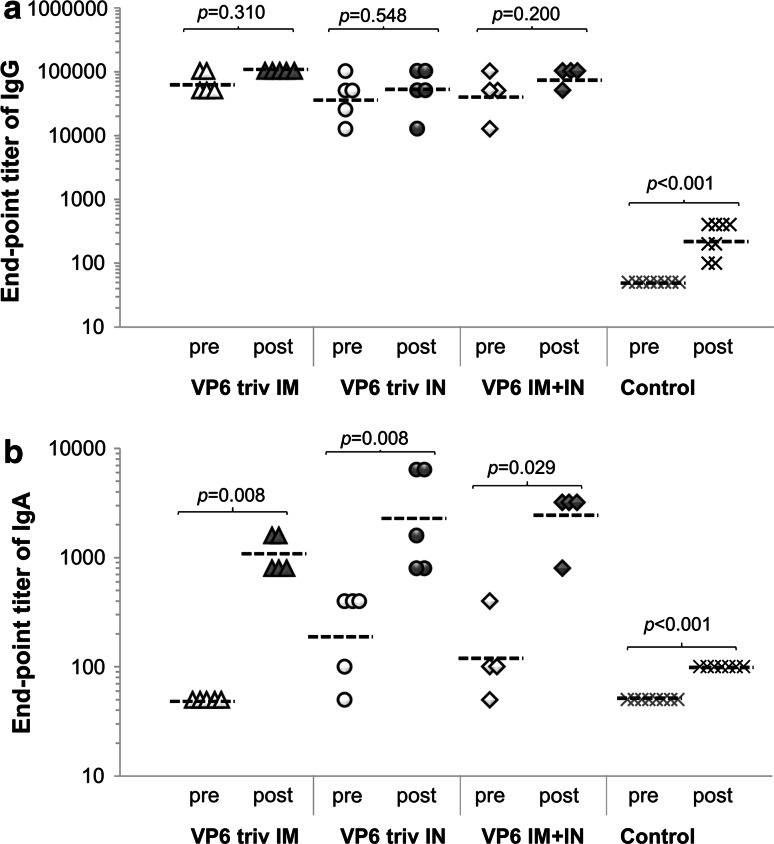
Pre- and post-challenge VP6-specific IgG (**a**) and IgA (**b**) antibodies in sera of individual mice immunized IM and IN with the trivalent vaccine containing rVP6 (5 mice/group) or sequentially IM+IN with rVP6 (4 mice/group). A sample was considered ELISA positive if the optical density at 490 nm (OD_490_) was above the set cutoff value (mean OD_490_ of control mice + 3 × SD) and ≥0.1. All control mice were combined (8 mice/group). Endpoint titers of individual mice, expressed as log_10_ of the reciprocal of the highest sample dilution giving a positive reading, as well as geometric mean titers of the groups (-----) at study weeks 5 (pre-challenge tail-blood sample) and 7 (post-challenge termination sera) are shown. A titer of 50 was assigned for all negative samples, being a half of the starting serum dilution. The statistical differences between non-parametric observations of independent groups were assessed by Mann-Whitney U-test (SPSS Inc, Chicago, IL); *p* ≤ 0.05 was considered to indicate a statistically significant difference

The quantity of RV antigen shed in fecal samples was determined up to 8 days post-challenge (Fig. 2a). A significant difference in viral shedding was detected between the mice immunized IM, IN and IM+IN and the control mice (*p* = 0.011), whereas the shedding between the immunized groups was not different (*p* = 0.514). The total antigen shedding of mice immunized IM and IN decreased 66 % (±12 %) and 65 % (±18 %) compared to the controls (Fig. 2a and b). Although sequential IM+IN immunization conferred a numerically higher protection rate (84 ± 5 %) (Fig. 2b), it was not statistically different from the groups immunized IM or IN.

**Fig. 2:**
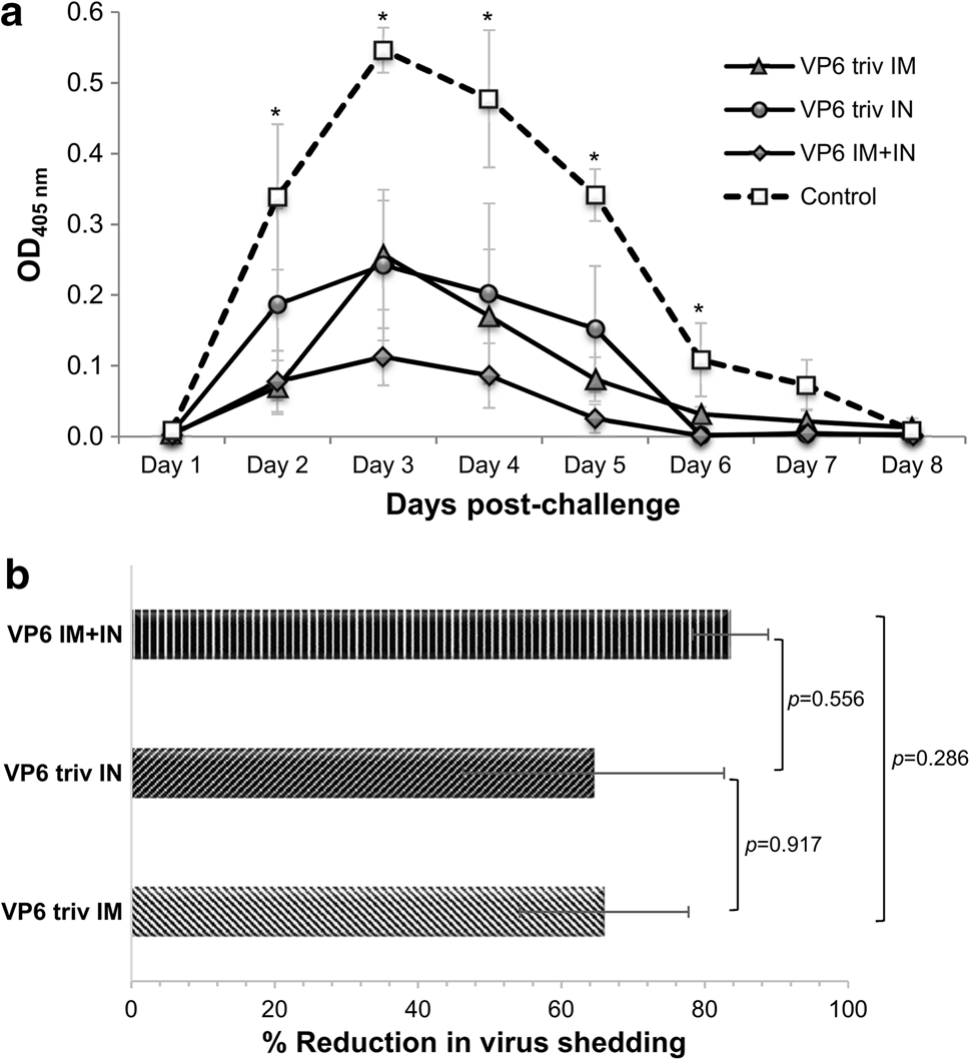
Protection against RV shedding in immunized mice. Viral shedding curves (OD_405_ versus day post-challenge) for each animal were plotted and the reduction in viral load was calculated by comparing the mean area under the shedding curve of the immunized mice to the mean area under the curve of the controls. **a.** Viral shedding curves of experimental groups. Each point represents the daily average of antigen shed per group with standard error of the mean. Asterisks (*) indicate a significant difference (*p* ≤ 0.05; Mann-Whitney U-test) in daily shedding between the immunized and control mice. **b.** Reductions in virus shedding of VP6-immunized mice following challenge. Mean percent reductions of the experimental groups with standard error of the means are shown. A >50 % reduction in virus shedding was considered significant protection from virus challenge, as reported previously

No correlation of pre-challenge titers of IgG (r = -0.455, *p* = 0.127) or IgA (r = -0.198, *p* = 0.497) antibodies with protection rates was detected. After the RV challenge, VP6-specific serum IgG and IgA antibody titers increased in all VP6-immunized mice (Fig. 1a and b), but only the levels of the post-challenge IgA increased significantly compared to the pre-challenge levels (*p* < 0.03). Protection levels correlated with the levels of serum IgA after the challenge (r = 0.607, *p* = 0.006). Following the challenge, control mice also developed low levels of IgG (GMT ≤ 2.5 log_10_) and IgA (GMT 2 log_10_), but the titers were significantly lower than those of the vaccinated mice (*p* < 0.001).

RV VP6 has been proposed as a subunit vaccine candidate against RV by us [14, 20, 21, 23] and others [17]. It forms different oligomeric structures *in vitro* [24], which are highly immunogenic in mice without the need for external adjuvants [14, 20, 21, 25]. Due to the repetitive multivalent antigenic structures, these oligomers are able to cross-link B-cell receptors very efficiently [26], whereas soluble VP6 generally requires an adjuvant for induction of an immune response [17]. Although the role of VP6 in protective immunity is still unclear, VP6 may be sufficient for protective immunity, as induction of protection against RV infection in mice and rabbits has been achieved with inactivated double-layered (dl) RV particles [27], dl2/6-VLPs [28] and VP6 protein [17, 21, 25] without the surface VP4 and VP7 antigens. Unlike the surface proteins, antibodies to the inner capsid VP6 are non-neutralizing. However, anti-VP6 IgA, but not IgG, is able to inhibit RV replication intracellularly [18, 29].

Human RV-derived rVP6 protein given parenterally or mucosally induced similar levels of protection against RV EDIM_wt_ infection. Protection was evaluated in an adult mouse model, which is an infection model but not a disease model, by measuring reduction in fecal RV antigen shedding after viral challenge [30]. Immunized mice showed significant reduction (>65 %) in virus shedding when compared to the controls. The protection was incomplete but of the order of magnitude that is achieved against any RV disease in humans after live RV vaccination. These results indicate efficacy of the rVP6-based vaccine in conferring protective immunity against live RV challenge independently of the delivery route. Similar reduction rates were previously published for mice immunized subcutaneously with rVP6 tubules [25]. Partial protection was also achieved with inactivated dl RV particles [27] and VP6 DNA vaccines after IM administration [31]. Protection close to 100 % against shedding of two murine RV strains has been elicited after IN immunization with MBP-VP6 only after inclusion of an external adjuvant [17].

Although intestinal IgA was shown to be critical for RV clearance and protection in the mouse model [32], serum RV IgA targeted to VP6 has been considered the best surrogate marker for vaccine-induced protection in humans [10, 11]. We detected a positive correlation between post-challenge VP6-specific serum IgA levels and the RV protection rate in mice. Both parenteral and mucosal delivery induced similar clearance of RV, even though only the IN and IM+IN routes led to detectable pre-challenge serum IgA antibodies. IM immunized mice may have had undetectable pre-existing serum IgA level, which expanded rapidly after viral replication in the gut [33]. Viral replication possibly led to a significant increase in serum IgA titers in VP6-primed mice, which correlated with reduction in RV antigen shedding and therefore protection. However, evidence of a correlation of serum IgA with protection has been contradictory in animal models [34]. By contrast, correlation of protection with serum IgA has been presented in mice following IN immunization with dl2/6-VLPs and cholera toxin [28].

In conclusion, the human RV rVP6 protein induced considerable protection in mice against live heterologous RV challenge, independently of the immunization route. These results highlight the importance of non-serotype-specific antibody responses induced using the highly conserved VP6 protein in heterotypic protection.

## References

[1] Yen C, Tate JE, Hyde TB (2014). Rotavirus vaccines: current status and future considerations. Hum Vaccin Immunother.

[2] Ruiz-Palacios GM, Perez-Schael I, Velazquez FR (2006). Safety and efficacy of an attenuated vaccine against severe rotavirus gastroenteritis. N Engl J Med.

[3] Vesikari T, Matson DO, Dennehy P (2006). Safety and efficacy of a pentavalent human-bovine (WC3) reassortant rotavirus vaccine. N Engl J Med.

[4] Zaman K, Dang DA, Victor JC (2010). Efficacy of pentavalent rotavirus vaccine against severe rotavirus gastroenteritis in infants in developing countries in Asia: a randomised, double-blind, placebo-controlled trial. Lancet.

[5] Madhi SA, Cunliffe NA, Steele D (2010). Effect of human rotavirus vaccine on severe diarrhea in African infants. N Engl J Med.

[6] Patel MM, Lopez-Collada VR, Bulhoes MM (2011). Intussusception risk and health benefits of rotavirus vaccination in Mexico and Brazil. N Engl J Med.

[7] Offit PA, Blavat G (1986). Identification of the two rotavirus genes determining neutralization specificities. J Virol.

[8] Desselberger U, Huppertz HI (2011). Immune responses to rotavirus infection and vaccination and associated correlates of protection. J Infect Dis.

[9] Angel J, Franco MA, Greenberg HB (2012). Rotavirus immune responses and correlates of protection. Curr Opin Virol.

[10] Patel M, Glass RI, Jiang B, Santosham M, Lopman B, Parashar U (2013). A systematic review of anti-rotavirus serum IgA antibody titer as a potential correlate of rotavirus vaccine efficacy. J Infect Dis.

[11] Cheuvart B, Neuzil KM, Steele AD (2014). Association of serum anti-rotavirus immunoglobulin A antibody seropositivity and protection against severe rotavirus gastroenteritis: analysis of clinical trials of human rotavirus vaccine. Hum Vaccin Immunother.

[12] Estes M, Kapikian A, Knipe D, Howley P, Griffin D, Lamb R, Martin M, Roizman B, Straus S (2007). Rotaviruses. Fields virology.

[13] Tang B, Gilbert JM, Matsui SM, Greenberg HB (1997). Comparison of the rotavirus gene 6 from different species by sequence analysis and localization of subgroup-specific epitopes using site-directed mutagenesis. Virology.

[14] Lappalainen S, Tamminen K, Vesikari T, Blazevic V (2013). Comparative immunogenicity in mice of rotavirus VP6 tubular structures and virus-like particles. Hum Vaccin Immunother.

[15] Svensson L, Sheshberadaran H, Vene S, Norrby E, Grandien M, Wadell G (1987). Serum antibody responses to individual viral polypeptides in human rotavirus infections. J Gen Virol.

[16] Esquivel FR, Lopez S, Guitierrez-X L, Arias C (2000). The internal rotavirus protein VP6 primes for an enhanced neutralizing antibody response. Arch Virol.

[17] Choi AH, McNeal MM, Basu M (2002). Intranasal or oral immunization of inbred and outbred mice with murine or human rotavirus VP6 proteins protects against viral shedding after challenge with murine rotaviruses. Vaccine.

[18] Burns JW, Siadat-Pajouh M, Krishnaney AA, Greenberg HB (1996). Protective effect of rotavirus VP6-specific IgA monoclonal antibodies that lack neutralizing activity. Science.

[19] Debbink K, Lindesmith LC, Donaldson EF, Baric RS (2012). Norovirus immunity and the great escape. PLoS Pathog.

[20] Tamminen K, Lappalainen S, Huhti L, Vesikari T, Blazevic V (2013). Trivalent combination vaccine induces broad heterologous immune responses to norovirus and rotavirus in mice. PLoS One.

[21] Lappalainen S, Pastor AR, Tamminen K (2014). Immune responses elicited against rotavirus middle layer protein VP6 inhibit viral replication in vitro and in vivo. Hum Vaccin Immunother.

[22] Malm M, Tamminen K, Vesikari T, Blazevic V (2015). Comparison of intramuscular, intranasal and combined administration of norovirus virus-like particle subunit vaccine candidate for induction of protective immune responses in mice. J Clin Cell Immunol.

[23] Blazevic V, Lappalainen S, Nurminen K, Huhti L, Vesikari T (2011). Norovirus VLPs and rotavirus VP6 protein as combined vaccine for childhood gastroenteritis. Vaccine.

[24] Lepault J, Petitpas I, Erk I (2001). Structural polymorphism of the major capsid protein of rotavirus. EMBO J.

[25] Pastor AR, Rodriguez-Limas WA, Contreras MA (2014). The assembly conformation of rotavirus VP6 determines its protective efficacy against rotavirus challenge in mice. Vaccine.

[26] Bachmann MF, Rohrer UH, Kundig TM, Burki K, Hengartner H, Zinkernagel RM (1993). The influence of antigen organization on B cell responsiveness. Science.

[27] McNeal MM, Rae MN, Conner ME, Ward RL (1998). Stimulation of local immunity and protection in mice by intramuscular immunization with triple- or double-layered rotavirus particles and QS-21. Virology.

[28] Siadat-Pajouh M, Cai L (2001). Protective efficacy of rotavirus 2/6-virus-like particles combined with CT-E29H, a detoxified cholera toxin adjuvant. Viral Immunol.

[29] Schwartz-Cornil I, Benureau Y, Greenberg H, Hendrickson BA, Cohen J (2002). Heterologous protection induced by the inner capsid proteins of rotavirus requires transcytosis of mucosal immunoglobulins. J Virol.

[30] Ward RL, McNeal MM, Sheridan JF (1990). Development of an adult mouse model for studies on protection against rotavirus. J Virol.

[31] Yang K, Wang S, Chang KO (2001). Immune responses and protection obtained with rotavirus VP6 DNA vaccines given by intramuscular injection. Vaccine.

[32] Blutt SE, Miller AD, Salmon SL, Metzger DW, Conner ME (2012). IgA is important for clearance and critical for protection from rotavirus infection. Mucosal Immunol.

[33] Coffin SE, Moser CA, Cohen S, Clark HF, Offit PA (1997). Immunologic correlates of protection against rotavirus challenge after intramuscular immunization of mice. J Virol.

[34] O’Neal CM, Harriman GR, Conner ME (2000). Protection of the villus epithelial cells of the small intestine from rotavirus infection does not require immunoglobulin A. J Virol.

